# Manual Collection and Semen Characterization in a West Indian Manatee (*Trichechus manatus*)

**DOI:** 10.3389/fvets.2020.569993

**Published:** 2020-10-22

**Authors:** Jonathan R. Cowart, Danielle M. Collins, Antonio A. Mignucci-Giannoni, Tamara Alejandro-Zayas, Antonio L. Rivera-Guzman, Iskande V. Larkin

**Affiliations:** ^1^Aquatic Animal Health Program, University of Florida College of Veterinary Medicine, Gainesville, FL, United States; ^2^Department of Animal Sciences, University of Florida, Gainesville, FL, United States; ^3^Caribbean Manatee Conservation Center, Inter American University of Puerto Rico, Bayamón, Puerto Rico; ^4^Center for Conservation Medicine and Ecosystem Health, Ross University School of Veterinary Medicine, Basseterre, Saint Kitts and Nevis

**Keywords:** manatee, CASA, reproductive physiology, semen characterization, sperm

## Abstract

Limited information is available regarding male reproductive physiology in West Indian manatees (*Trichechus manatus*). Currently, only basic ultrastructural and morphometric descriptions of the spermatozoon exist; however, there are no reports evaluating any seminal characteristics in this species. Therefore, the aim of the study was to fill current gaps in knowledge regarding semen parameters in West Indian manatees by collecting and characterizing multiple ejaculate samples from a single, adult West Indian manatee. Samples were analyzed for the following semen parameters: volume, agglutination, pH, osmolality, viscosity, concentration, total sperm number, motility and kinematic parameters, morphology, plasma membrane integrity, acrosome integrity, chromatin maturation, and chromatin condensation. All macroscopic semen parameters varied to some extent between samples. Total and progressive motility was high for ejaculates 2 to 5, exceeding 97 and 89%, respectively; however, these parameters decreased dramatically throughout ejaculates 6 and 7. Across all samples, curvilinear velocity, straight-line velocity, and average pathway velocity represented the largest significant differences (*p* < 0.001) between each of the progression velocity subgroups (rapid, medium, slow). Sperm characteristics, including acrosome integrity (79.8%), chromatin condensation (93.1%), and chromatin maturation (99.5%) were very high; however, high numbers of morphologically abnormal sperm were present (52.9%) and plasma membrane integrity was low (45.1%). These results are the first of their kind for this species and suggest high semen quality, based on multiple ejaculates, in this male West Indian manatee.

## Introduction

The West Indian manatee (*Trichechus manatus*) is one of the most well-studied sirenian species. The geographic range of this species includes the coastal southeastern United States, particularly Florida, Mexico through Central America, the Greater Antilles of the Caribbean, and the northern coasts of Colombia and Venezuela to eastern Brazil (1, 2). Throughout its range, this species faces a myriad of anthropogenic and natural threats including watercraft collisions (3–5), entanglement and ingestion of marine debris (4, 6), brevetoxicosis from annual harmful algal blooms (red tide) (7, 8), cold stress syndrome (9), and low genetic variability (10–12), which all contribute to high annual injury and mortality.

Because of the current threats this species faces, it is essential to understand different life history parameters for effective management and conservation. Knowledge regarding reproductive biology is critical and has implications for continued population survival and growth. Increased understanding of West Indian manatee reproductive physiology has not been adequately represented in the expansive growth of sirenian research. Unlike other marine mammals managed in zoological institutions and aquaria, such as the bottlenose dolphin (*Tursiops truncatus*), orca (*Orcinus orca*), and beluga (*Delphinapterus leucas*), no captive breeding program exists for manatees in the United States (U.S.), further limiting opportunities to better understand the reproductive physiology of this species. High investment in reproduction and slow reproductive rates are limiting factors for the continued growth of manatee populations and without a complete understanding of reproduction in this species, long-term management and conservation of this species is potentially at risk.

Previous studies on reproduction in male West Indian manatees are limited to basic ultrastructural and morphometric descriptions of the spermatozoa (13), seasonal and developmental changes in spermatogenesis (14), size of the testes and seminal vesicles in relation to sperm competition (15), limited hormonal monitoring (16, 17), and most recently gross anatomical and histological descriptions of the reproductive tract (18, 19). While these studies provide valuable information, many gaps still remain and no information on semen parameters is available for this, or any other sirenian species. Therefore, the goal of this study was to characterize semen parameters in multiple ejaculates from a single, captive male West Indian manatee to better understand the reproductive biology of this species and build a foundation of baseline data regarding semen parameters for future reproductive research.

## Materials and Methods

### Animal

Semen was collected from a wild-born, non-releasable adult West Indian manatee (estimated age 11 years old, total length 289 cm, weight 340 kg) under managed care at the Caribbean Manatee Conservation Center, Inter American University in Bayamón, Puerto Rico. This manatee represents the only male manatee trained for semen collection in the entire United States and accompanying territories. The male was deemed to be clinically healthy at the time of collections and exhibited no clinical or subclinical health issues during the sample collection period. The male was housed alone in an outdoor 7.4 m diameter octagonal pool holding ~91,200 L of fresh water maintained at an average temperature of 27.9°C. The manatee was fed a diet consisting of a mix of vegetables including romaine and iceberg lettuce, spinach, cabbage, sweet potatoes, apples, bananas, honeydew, cantaloupe, and primate leaf-eater monkey chow. Ejaculate samples were collected as part of comprehensive veterinary health assessments and shared for use in this study. All samples were received and analyzed under Federal Fish and Wildlife Permit #MA067116-2 and in accordance with Inter American University and University of Florida Institutional Animal Care and Use Committees (UF IACUC protocol #20180884).

### Semen Collection

**Figure 1:**
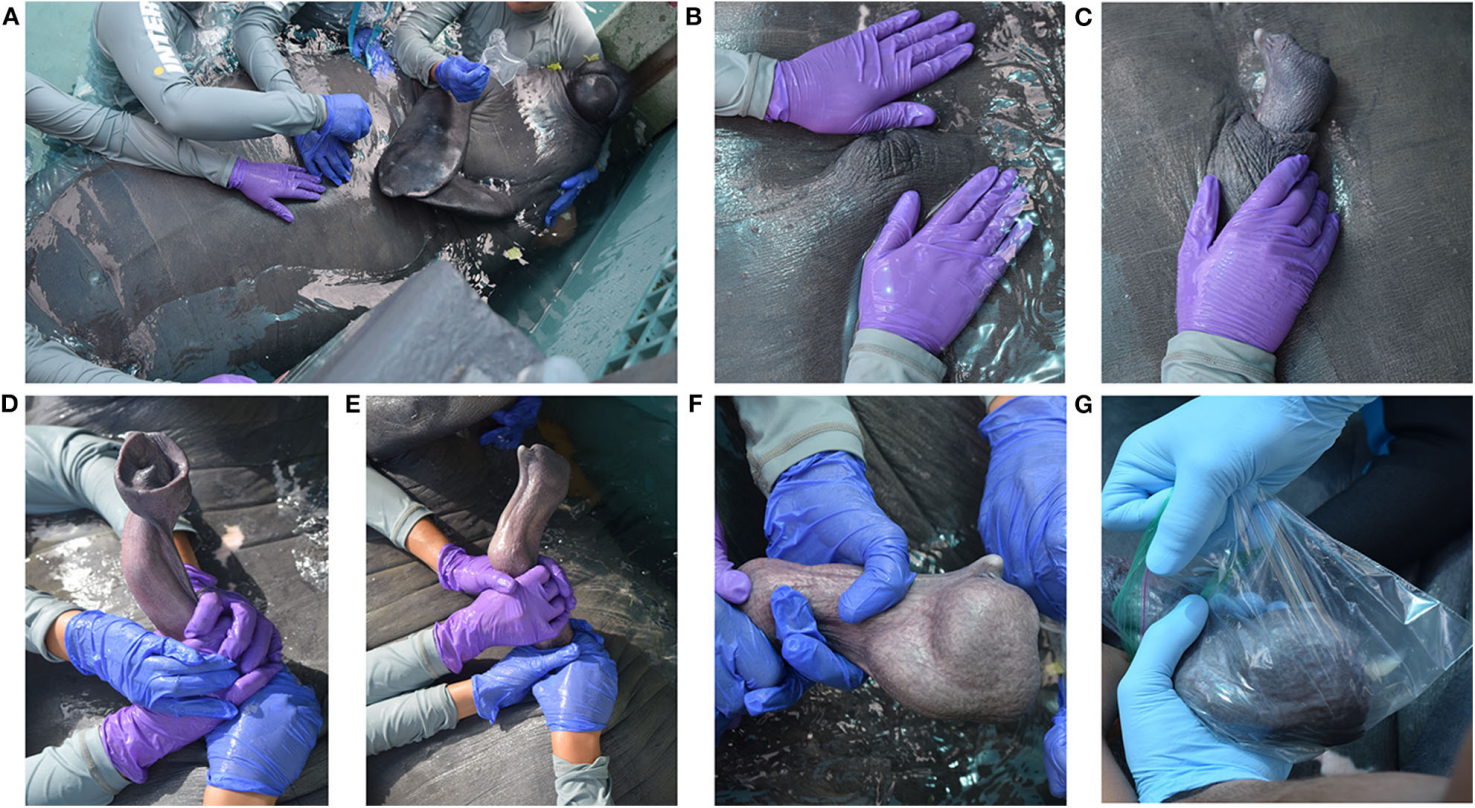
Manual semen collection in a captive male West Indian manatee. **(A)** The male is positioned at the edge of the pool with his ventral side above the water and his body supported by in-water trainers. **(B,C)** The male receives tactile stimulation to the genital region until partial extrusion or a full erection is achieved. Tactile stimulation is focused on the mid-shaft region and glans penis to elicit the best stimulatory response. **(D,E)** The penis enters a pre-ejaculatory state characterized by the formation of a penile cup at the distal end of the penis and a bulbis glandis along the midshaft of the penis. **(F)** The penile cup becomes increasingly engorged as the male approaches ejaculation. **(G)** Upon ejaculation, semen is collected into a clear plastic bag for immediate processing and analysis.

Semen collection was attempted once a day, at approximately the same time each morning, for a 2-week period in March 2018. The male was trained through operant conditioning to position at the edge of the pool and roll over with its ventral side above the water where his body was further supported by facility trainers (Figure 1A). The male received tactile stimulation to the genital region until arousal (partial extrusion or erection of the penis) was accomplished (Figures 1B,C). Upon full penile extrusion, manual stimulation was performed until ejaculation was achieved. Semen collection times ranged between 10 and 30 min from the start of genital stimulation to ejaculation. Positive reinforcement techniques were used upon ejaculation to strengthen the association between the behavior and desired outcome. Manual stimulation was focused upon the mid-shaft and glans regions of the penis as the combination of these areas elicited the best stimulatory response. A pre-ejaculatory state could be recognized by both a distinct flaring of the glans penis, which resulted in the formation of a penile cup, and an apparent bulb of erectile tissue along the midshaft of the penis, likely homologous to the bulbis glandis of other species (Figures 1D,E). The penile cup quickly became increasingly engorged as the male approached ejaculation (Figure 1F). At ejaculation, semen was collected into a clean plastic bag and transported to the laboratory for immediate evaluation (Figure 1G). In the laboratory, semen was transferred to 15 ml tubes and kept at 37°C for the duration of the analyses. All samples were processed and analyzed within 30 min of collection.

### Macroscopic Semen Characteristics

Ejaculate samples were analyzed using standard protocols for the following macroscopic semen characteristics: volume, color, pH, osmolality, viscosity. The pH of each ejaculate was measured using pH indicator strips (Ricca Chemical Company, Arlington, TX) and osmolality (mOsm) was analyzed using a vapor pressure osmometer (VAPRO; Wescor Inc., Logan, Utah). Osmolality was measured in triplicate and reported as the mean value for each ejaculate sample. Viscosity was analyzed using a 20 μm deep four-chambered slide (Cell Vision Technologies, Heerhugowaard, The Netherlands) and measuring the elapsed time for the sample to traverse the chamber.

### Microscopic Sperm Characteristics

#### Agglutination, Concentration, and Total Sperm Number

Agglutination was assessed using a sperm agglutination rating (0–5 scale) as described by O'Brien et al. (20), where 0 represents no agglutination and 5 represents 100% agglutination. Concentration and total sperm number were determined using an Improved Neubauer Brightline hemacytometer.

#### Motility and Kinematic Parameters

Sperm motility and kinematics were objectively assessed using the Sperm Class Analyzer^®^ (SCA^®^) version 6.3 computer-aided sperm analysis (CASA) system (Microptic S.L., Barcelona, Spain). Slides were viewed with the use of a Basler aCa 1300–200uc digital camera mounted on a Nikon Eclipse E200 LED microscope equipped with a portable heating stage. Motility parameters were captured with 50 frames at a frame rate of 50 frames/second. Total motility, progressive motility (including rapid progressive, medium progressive, and slow progressive), non-progressive, and immotile motility parameters were analyzed for each sample. Progressive motility was defined as straightness (STR) >75. Kinematic parameters analyzed for each sample included: curvilinear velocity (VCL, μm/s), straight-line velocity (VSL, μm/s), average pathway velocity (VAP, μm/s), linearity (LIN, %), straightness (STR, %), wobble (WOB, %), amplitude of lateral head displacement (ALH, μm), and beat cross frequency (BCF, Hz). Differentiation between velocity subgroups (rapid, medium, slow) was determined using default SCA^®^ settings for mammals (selected animal: stallion) with VCL cut-off values of 25 <45>90 μm/s. Each sample was evaluated using a flush technique as described by Boshoff et al. (21) where 1.5 μl semen was pipetted into a pre-warmed (37°C), 20 μm deep four-chambered slide and “flushed” with 1.5 μl pre-warmed phosphate buffered saline (PBS). Five randomly-selected microscopic fields were analyzed to calculate each motility and kinematic parameter.

#### Plasma Membrane Integrity and Morphology

Sperm plasma membrane integrity and morphology were assessed by mixing 10 μl of semen with 40 μl of an eosin-nigrosin stain (BrightVit; Microptic S.L., Barcelona, Spain) for 5 min. A smear was made, air-dried overnight, and permanently mounted with Eukitt mounting medium (Electron Microscopy Sciences, Hatfield, PA). Sperm were classified as having an intact membrane (viable) if no stain uptake by the sperm head was visible and having a non-intact membrane (non-viable) if there was partial or complete stain uptake by the sperm head (1,000 sperm per sample, 400x total magnification). For morphological assessment, sperm were visually assessed for structural abnormalities including head abnormalities, bent or coiled midpieces, cytoplasmic droplets, bent or coiled tails, and detached heads (200 sperm per sample, 1000x total magnification).

#### Acrosome Integrity

Sperm acrosome integrity was evaluated using a modified Coomassie blue staining procedure as described by Larson and Miller (22). Fixed sperm were washed twice in 100 mM ammonium acetate and the sperm pellet resuspended in 100 mM ammonium acetate. A 20 μl aliquot of the sperm sample was smeared on a glass slide and air-dried. Air-dried slides were stained in Coomassie blue (0.22% Coomassie blue G-250, 50% methanol, 10% acetic acid, 40% water), for 4 min, briefly rinsed in distilled water, air-dried, and permanently mounted with Eukitt mounting medium. For acrosome integrity, sperm were categorized as having either an intact acrosome or a non-intact acrosome (200 sperm per sample, 400x total magnification). An intact acrosome stained dark blue with a distinct outline while non-intact acrosomes were either partially or completely lost.

#### Chromatin Integrity

**Figure 2:**
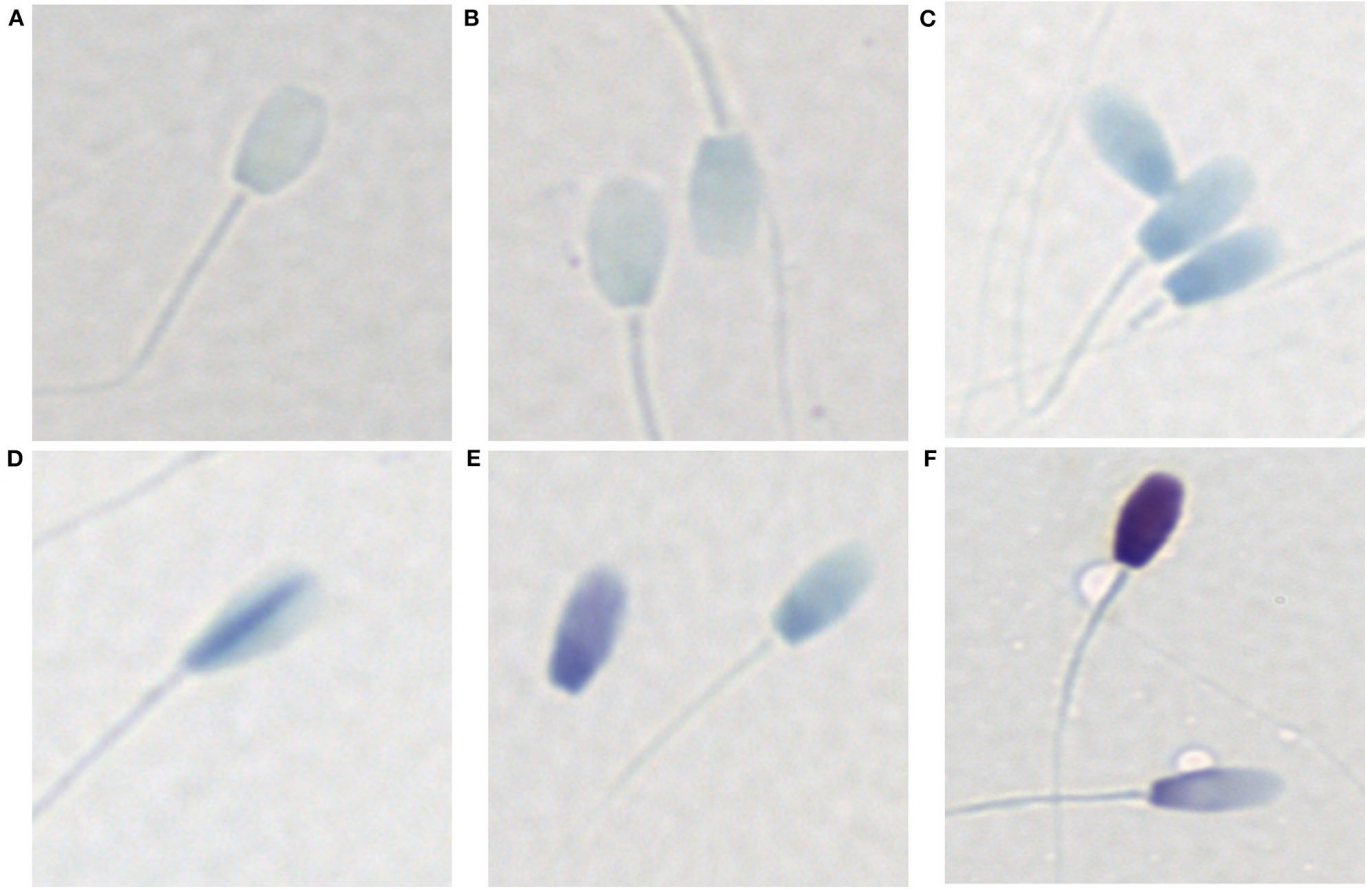
Toluidine blue staining patterns associated with varying levels of chromatin condensation in the West Indian manatee. **(A)** no stain uptake, **(B)** very light pale blue (very slight stain uptake with bipolar orthochromatic staining), **(C)** light pale blue (slightly darker stain uptake with more defined bipolar orthochromatic staining), **(D)** dispersive dark blue (mostly light blue with dark blue dispersed, generally creating a line up the middle of the sperm head), **(E)** intermediate blue/violet (either darker blue or a light violet/purple color with or without bipolar staining), **(F)** dark blue/violet (intense blue/violet metachromatic staining). **(A-C)** are considered normal with normal chromatin condensation, **(D–F)** represent abnormal chromatin condensation with varying levels of chromatin decondensation.

**Figure 3:**
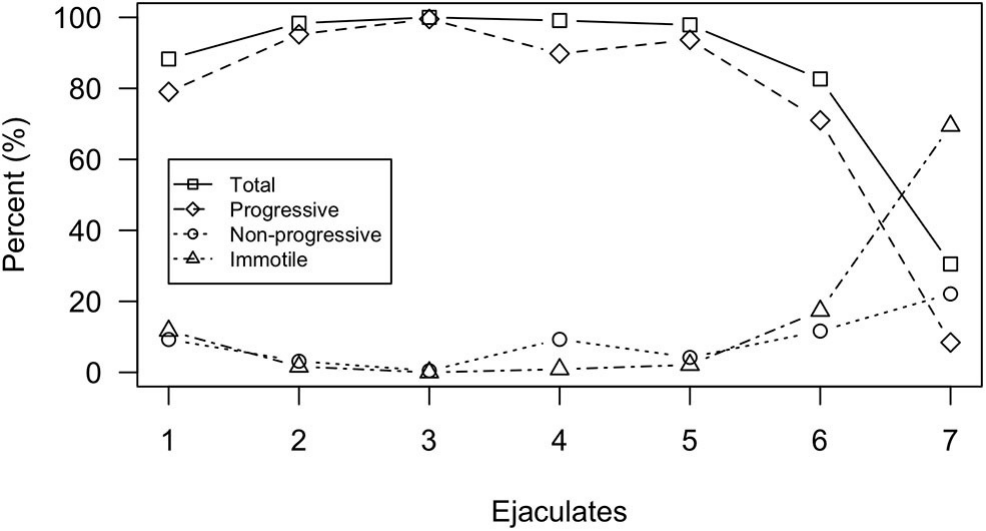
Motility parameters analyzed by the SCA^®^ system for each individual ejaculate sample. Motility parameters include total motility, progressive motility, non-progressive motility, and immotility.

**Figure 4:**
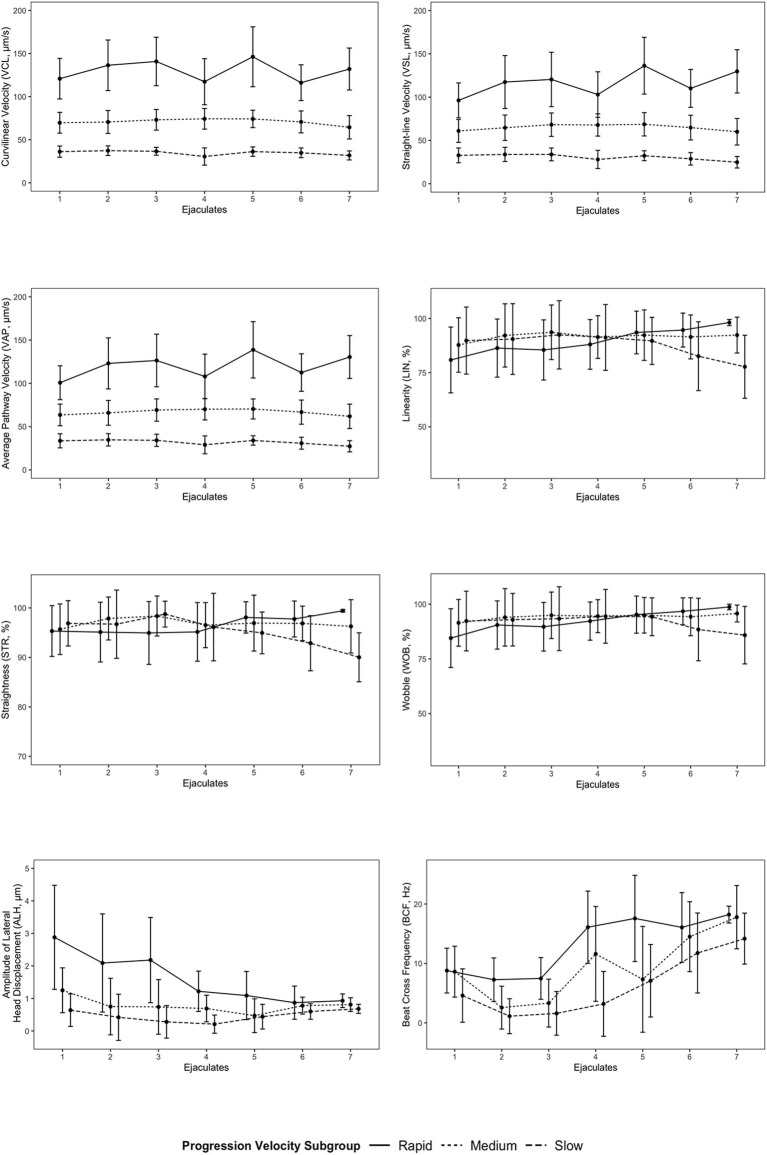
Mean values of progression velocity subgroups for each kinematic parameter for each individual ejaculate sample. Error bars represent standard deviation. Points for progression velocity subgroups for each ejaculate sample are offset for linearity (LIN), straightness (STR), wobble (WOB), amplitude of lateral head displacement (ALH), and beat cross frequency (BCF) to provide visual clarity between each subgroup.

Sperm chromatin condensation was assessed with a toluidine blue staining protocol as described by Kim et al. (23). Sperm were washed twice in PBS and the sperm pellet resuspended in PBS. A 10 μl aliquot of sperm was smeared on a glass slide and air-dried overnight. Air-dried slides were fixed in 95% ethanol-acetone (1:1) at 4°C for 1 h and air-dried overnight. Slides were then hydrolyzed in 0.1 N HCl at 4°C for 5 min, rinsed in distilled water, stained in 0.05% toluidine blue (in 50% McIlvaine citrate phosphate buffer) for 5 min, rinsed briefly in distilled water, air-dried overnight and permanently mounted with Eukitt mounting medium. Six levels of toluidine blue staining were differentiated for the West Indian manatee: (1) no stain uptake (Figure 2A), (2) very light pale blue (very slight stain uptake with bipolar orthochromatic staining) (Figure 2B), (3) light pale blue (slightly darker stain uptake with more defined bipolar orthochromatic staining) (Figure 2C), (4) dispersive dark blue (mostly light blue with dark blue dispersed, mostly creating a line up the middle of the sperm head) (Figure 2D), (5) intermediate blue/violet (either darker blue or a light violet/purple color with or without bipolar staining) (Figure 2E), (6) dark blue/violet (intense blue/violet metachromatic staining) (Figure 2F). Levels 1-3 were considered normal with normal chromatin condensation, level 4 was considered an intermediate with slight chromatin decondensation, and levels 5–6 were considered abnormal with compromised chromatin condensation (200 sperm per sample, 400x total magnification).

Sperm chromatin maturity, or nuclear maturation, was assessed with a modified aniline blue-eosin staining protocol as described by Wong et al. (24). Sperm smears were prepared in the same manner as that for toluidine blue staining. Air-dried slides were fixed in 4% formalin for 5 min at room temperature and air-dried overnight. Slides were rinsed in distilled water and stained in a 5% aniline blue in 4% acetic acid solution for 5 min. After staining with aniline blue, slides were rinsed in distilled water, and then stained in 0.5% eosin for 1 min. Stained slides were rinsed in distilled water, air-dried overnight, and permanently mounted with Eukitt mounting medium. Immature sperm stained dark blue while mature sperm stained red-pink by the eosin counterstain (200 sperm per sample, 400x total magnification).

### Statistical Analysis

Macroscopic and microscopic data were characterized by descriptive statistics and are presented as mean and standard deviation (mean ± *SD*). Kinematic and progression velocity subgroup data were group-centered and then tested for normality using the Shapiro-Wilk test. Because the data met the assumptions for normality, kinematic and progression velocity subgroup data were analyzed using a one-way ANOVA followed *post hoc* with Tukey's Honestly Significant Differences for multiple pairwise comparisons. *p* < 0.05 was considered significant. All statistical analyses were completed using R software version 3.6.1.

## Results

### Macroscopic Semen Characteristics

A total of seven ejaculates were collected over the 2-week sampling period with urine contamination occurring in two of the ejaculate samples. The values for all macroscopic semen parameters analyzed for each ejaculate sample are summarized in Table 1. Ejaculate volume varied between samples ranging from 26 ml-45 ml ($\bar{\text{x}}$ = 37.3 ml). pH was consistent for all samples except for ejaculates 3 and 4, which were urine contaminated. Semen osmolality ranged from 282 mOsm-300 mOsm ($\bar{\text{x}}$ = 292 mOsm). Viscosity was highest for ejaculate 1, but remained consistent for subsequent samples.

**Table 1:** Macroscopic and microscopic characteristics of each ejaculate sample.

**Ejaculate**	**Date**	**Total Volume (ml)**	**Sperm concentration (×10^**6**^/ml)**	**Total number of sperm (×10^**9**^)**	**Viscosity (sec)**	**Osmolality (mOsm)**	**pH**	**Agglutination**	**Color/appearance**	**Comments**
1	28 Feb	45	97.5	4.4	8.8	292	8.5	3	Moderately translucent	
2	01 Mar	44	201	8.8	–	290	8.5	1	Milky white/slightly translucent	
3	02 Mar	39	99.8	3.9	4.4	282	8.5–9.0	1	Moderately translucent	Urine contaminated
4	05 Mar	26	71.9	1.9	4.3	300	8.0–8.5	2	Milky white	Urine contaminated
5	06 Mar	44	70	3.1	4	288	8.5	–	Moderately translucent	
6	08 Mar	30^*^	9.8^*^	0.3^*^	4.4	300	8.5	1–2	Moderately translucent with agglutinated clumps	
7	09 Mar	33	21	0.7	4.9	290	8.5	3	Moderately translucent	
Mean ± *SD*		37.3 ± 7.7	81.6 ± 61.1	3.3 ± 2.9	5.1 ± 1.8	292 ± 8	8.5	2		
Range		26–45	9.8–201	0.3–8.8	4–8.8	282–300	8.3–8.8	1–3		

* *Values are slightly lower than actual values as some of the sample was lost during collection. These values represent the amount that was collected, not including what was lost*.

### Microscopic Sperm Characteristics

#### Agglutination, Concentration, and Total Sperm Number

Values for agglutination, concentration, and total sperm number for each ejaculate sample are summarized with the macroscopic semen characteristics in Table 1. Agglutination, which was highest in ejaculates 1 and 7, displayed both head-to-head and tail-to-tail agglutination types in each of the ejaculate samples. Concentration was highly variable between samples with a generally declining trend over the sample collection period. Total sperm number was also highly variable between samples ($\bar{\text{x}}$ = 3.3 ×10^9^) with ejaculate 2 having the highest total sperm number of all ejaculate samples.

#### Sperm Motility and Kinematics

Sperm motility and kinematic data are summarized in Table 2 and Supplementary Tables 1, 2. Sperm motility (total and progressive) was high (>80% total motility) for ejaculates 1–6, despite urine contamination occurring in ejaculates 3 and 4, but decreased dramatically by the end of the sampling period with total motility equaling 30.5% for ejaculate 7 (Figure 3). Motility parameters were highest for ejaculates 2–5 with total motility exceeding 97% and progressive motility exceeding 89%. Mean total and progressive motility across all ejaculate samples was 85.3 and 76.7%, respectively. If excluding ejaculate 7 as an outlier, mean total motility increased from 85.3 to 94.4% and progressive motility increased from 76.7 to 88.1%.

**Table 2:** Motility and associated kinematic parameters across all ejaculate samples (*n* = 7).

**Motility Parameters**	**Mean ± *SD***	**Range**
**General Motility Characteristics**		
Total motility (%)	85.3 ± 25	30.5–100
Progressive motility (%)	76.7 ± 31.7	8.4–99.5
Non-progressive motility (%)	8.6 ± 7.2	0.5–22.1
Immotile (%)	14.7 ± 25	0–69.5
**Progressive Motility Kinematic Characteristics**		
**VCL (μm/s)**		
Rapid	132 ± 30^a^	90.1–388.7
Medium	72.4 ± 12.3^b^	45.1–90
Slow	33.3 ± 8^c^	11.9–44.9
**VSL (μm/s)**		
Rapid	114.5 ± 30.7^a^	28–359.1
Medium	65.8 ± 13.6^b^	19.9–89.9
Slow	29.2 ± 9.1^c^	4–44.3
**VAP (μm/s)**		
Rapid	119.8 ± 30^a^	30.7–359.7
Medium	67.9 ± 13.1^b^	20.3–89.9
Slow	30.6 ± 8.7^c^	4–44.3
**LIN (%)**
Rapid	87 ± 13.1	24.3–100
Medium	91.1 ± 11.6	26.2–100
Slow	87.4 ± 16	27.3–100
**STR (%)**
Rapid	95.3 ± 6	75–100
Medium	96.8 ± 4.6	75.2–100
Slow	94.9 ± 6.3	75.3–100
**WOB (%)**		
Rapid	91 ± 10.5	25.9–100
Medium	93.9 ± 9.5	26.7–100
Slow	91.5 ± 13.4	30.2–100
**ALH (μm)**		
Rapid	1.8 ± 1.3^a^	0–9.2
Medium	0.8 ± 0.6^b^	0–3.8
Slow	0.4 ± 0.4^b^	0–2
**BCF (Hz)**		
Rapid	10.7 ± 6.7	0–37
Medium	9.2 ± 7.4	0–31.8
Slow	6.6 ± 7.1	0–26

*Values with different superscripts within the same parameter are significantly different (p < 0.001)*.

Across all ejaculates, the proportion of sperm composing each progression velocity subgroup was highest for rapid progressive ($\bar{\text{x}}$ =61.3 ± 29.6%) followed by medium progressive ($\bar{\text{x}}$ =24.7 ± 17.8%) and slow progressive ($\bar{\text{x}}$ =14 ± 24%), respectively. Only ejaculates 1 and 7 differed from this trend. For ejaculate 1, medium progressive had the highest proportion of sperm (57.7%) followed by rapid progressive (35.5%) and slow progressive (6.8%). For ejaculate 7, slow progressive had the highest proportion of sperm (67.2%) followed by medium progressive (19%) and rapid progressive (13.8%).

Progression velocity subgroups for some kinematic parameters differed across all ejaculate samples (Table 2). VCL, VSL, and VAP were significantly different (*p* < 0.001) between each of the progression velocity subgroups (rapid, medium, slow). The rapid progression velocity subgroup was significantly different than both medium and slow progression velocity subgroups for ALH, but there was no significant difference between the medium and slow progression velocity subgroups. There were no significant differences between the progression velocity subgroups for LIN, STR, WOB, and BCF. Mean values of progression velocity subgroups for each kinematic parameter for each individual ejaculate sample are shown in Figure 4.

#### Plasma Membrane Integrity, Morphology, Acrosome Integrity, and Chromatin Integrity

Sperm integrity characteristics are summarized in Table 3 and Supplementary Tables 3, 4. Sperm plasma membrane integrity was relatively low ($\overset{-}{\text{x}}=$ 45.1%), compared to the number of live motile sperm, and the number of sperm with interrupted plasma membranes was generally greater than the percentage of immotile sperm. Plasma membrane integrity only exceeded 50% for ejaculates 2 and 3 (58.4 and 56.6%, respectively). Mean normal morphology across all samples was 47.1%. High numbers of morphologically abnormal sperm ($\overset{-}{\text{x}}=$ 52.9%) were present in each sample. Bent or coiled tails ($\overset{-}{\text{x}}=$ 20.1%) represented the most common morphological abnormality with detached heads ($\overset{-}{\text{x}}=$ 16%) and bent midpieces ($\overset{-}{\text{x}}=$ 12%) accounting for the other common abnormal morphologies. Mean acrosome integrity was 79.8% for all samples with ejaculates 2, 3, and 7 >80% (87, 86, and 82.5%, respectively). The proportion of sperm with normal chromatin condensation was very high across all ejaculates ($\overset{-}{\text{x}}=$ 93.1%) with low levels of chromatin decondensation occurring in each sample ($\overset{-}{\text{x}}=$ 6.9%). The highest levels of chromatin decondensation occurred in ejaculates 5 and 6 (14 and 11.5%, respectively). Similarly, chromatin maturity was exceptionally high across all ejaculates ($\overset{-}{\text{x}}=$ 99.9%), with ejaculate 5 the only sample exhibiting any level of chromatin immaturity ($\overset{-}{\text{x}}=$ 0.5%).

**Table 3:** Sperm characteristics across all ejaculate samples.

**Sperm Characteristics**	**Mean ± *SD***	**Range**
Plasma membrane integrity	45.1 ± 10.3	36.7–58.4
Acrosome integrity	79.8 ± 5.7	74–87
Chromatin condensation	93.1 ± 4.3	86–98
Chromatin maturation	99.9 ± 0.2	99.5–100
**Morphology**		
Normal	47.1 ± 12.8	21.5–57.5
Abnormal head	1.9 ± 0.8	0.5–3
Detached head	16 ± 8.9	7–32
Proximal cytoplasmic droplet	1.1 ± 0.9	0–2
Distal cytoplasmic droplet	1.4 ± 1.1	0–3
Abnormal midpiece	12 ± 3.2	7.5–15.5
Coiled/bent tail	20.1 ± 8.1	7–30

*All values are presented as percentages*.

## Discussion

This study represents the first semen characterization and application of computer-aided sperm analysis (CASA) in any sirenian species. Likewise, the current data comprise the only information available on any semen characteristic for this species. While some parameters were considered very good (motility, acrosome integrity, chromatin condensation, and chromatin maturation), other parameters were moderate to poor (plasma membrane integrity and morphology). It should be noted that plasma membrane integrity may be artificially low based on the eosin-nigrosin method that was utilized in this study. The number of “live” sperm were considerably lower than the number of motile sperm for each ejaculate sample, which may be due to the elapsed time between staining and observation. Future research should investigate optimal staining to observation times for the eosin-nigrosin method in this species in addition to exploring other techniques such as hypo-osmotic swelling test (HOS-test) and use of fluorochromes. Regardless though, these data suggest that manatee semen, or at the very least, semen from this individual male, is of good quality overall.

Ejaculate volumes recorded in this study were lower than expected considering the large size of the accessory sex glands (seminal vesicles and prostate) in this species. Postmortem assessment of the seminal vesicles from Florida manatees (*Trichechus manatus latirostris*), a subspecies of the West Indian manatee, shows that these glands are quite large and generally contain a volume of seminal fluid that far exceeds any ejaculate volume recorded in this study, especially during the breeding season (25) The low ratio of ejaculate volume to accessory sex gland size may reflect the mating strategy of males and could potentially ensure the capability for multiple ejaculates of acceptable quality over short periods of time when a female is receptive for mating. While ejaculate volumes were lower than expected for this species, they are relatively higher in comparison to recorded ejaculate volumes of other marine mammal species such as the orca [13.9 ml, (26)] and bottlenose dolphin [20.4 ml, (27)]. This is potentially due to the presence of seminal vesicles in manatees, which are absent in cetaceans. However, ejaculate volume was significantly lower than values recorded for Asian elephants [98.5 ml, (28)], an evolutionarily-related species.

While the data from this study add to our knowledge of male manatee reproductive physiology, it must be interpreted with caution. As previously noted, no data for any semen characteristic exists for any sirenian species, thereby precluding the establishment of normal reference ranges for any of the parameters analyzed in this study. Likewise, these samples are from a single male, thus, generalizations at a population level are unwarranted and limited inferences can be made. Additional ejaculate samples from other manatees are necessary to establish baseline data for semen parameters in this species. Unfortunately, no other manatees are currently trained for this type of sample collection. In the U.S., most non-releasable male manatees under managed care are trained for urine sampling and there is common concern that these manatees will be unable to differentiate between urine and semen operant conditioning signals and collection procedures. While there is an understandable basis for this concern, the stimulatory nature of the manual semen collection described in this study, coupled with appropriate husbandry training, should elicit the necessary autonomic responses involved with ejaculation, thereby allowing differentiation between urine and semen collection.

Continued refinement and optimization of semen collection protocols are necessary for future sampling and should incorporate techniques established in other marine mammal species. In this study, some issues were experienced during semen sample collection including frequent losses of erection (partial or full retraction of the penis) and urine contamination. Loss of erection was experienced most often when the male entered a pre-ejaculatory state and the glans of the penis appeared most tactilely sensitive. If full retraction of the penis occurred, tactile stimulation of the genital area was performed until arousal was achieved and manual stimulation could be reinitiated. Urine contamination was encountered during 2 sampling days and was determined by a drastic loss of initial sperm motility, changes in semen pH, and the presence of urine crystals. The occurrence of urination during sampling periods seemed to be due to normal contractions of the body exhibited during penile stimulation. This was corrected in subsequent sampling days by releasing the male from the procedure after multiple contractions were experienced and straining was evident. Urination was confirmed by an in-pool trainer and the sampling protocol was reinitiated afterwards until the sample was collected.

Use of an artificial vagina similar to that described for dolphins (20) or one more representative of the female manatee reproductive tract would be useful for collections in manatees to facilitate semen collection of maximum quality. As described in other species, ejaculates produced during intercourse have superior characteristics, including increased volume and total sperm number, greater percentage of morphologically normal sperm, and enhanced motility parameters, in comparison to those produced during masturbation (29–31). The enhanced quality of ejaculates produced during intercourse may be contributed to greater intensity and/or duration of sexual arousal, which generally exceeds that attained during masturbation (32). In manatees, the effects of sexual stimulation are unknown, but it can be assumed that greater levels of arousal and increased duration of sexual stimulation can be achieved with the use of an artificial vagina and this will positively influence the characteristics of each ejaculate. This may also help to reduce the number of erectile losses by reducing or eliminating the possible negative tactile feeling experienced when the collection bag was placed over the distal end of the penis when the male was in a pre-ejaculatory state.

Many semen parameters varied between sampling days, with some parameters (volume, concentration, and osmolality) decreasing with each successive sample day, only increasing again when single-day breaks were introduced. The opposite was true for motility parameters (total and progressive; except for ejaculates 6 and 7), which tended to increase with each successive sample day, only decreasing after single-day breaks. However, because this is the only male for which semen parameters have been measured, it is impossible to interpret beyond an individual level. Variation in ejaculate quality is well-documented in other mammalian species with some species, such as the bottlenose dolphin (33, 34), increasing semen quality characteristics with successive ejaculations while other species, such as the angora goat (*Capra hircus ancryrensis*) (35) and Pleven blackhead ram (*Ovis aries*) (36), decrease semen quality characteristics with successive ejaculations. However, these studies included multiple ejaculations during collection days, which was not the case for the male in our study where only a single collection was attempted per day. Attempted collections on an everyday basis may be a contributing factor to the decreases in motility and other parameters that were seen toward the end of the study period. A number of ejaculations in such close succession may or may not be physiologically representative of reproduction in wild populations. Future research should investigate the effect of time between ejaculation and overall semen quality.

Overall, while interpretation of the data in this study is limited due to animal sample size, this study provides the first data of its kind on male manatee semen parameters. Furthermore, this study provides a solid foundation for further reproductive research and the potential future application of assisted reproductive techniques (ART) in this species. Future studies should focus on semen characterization from additional males over extended time periods in order to establish normal reference values for this species. Additionally, the potential effects of environmental conditions, season, and reduced genetic heterozygosity on semen parameters in this species needs to be investigated. The continued study of the reproductive physiology of West Indian manatees represents an essential component of the species management plan and is critical for its overall conservation.

## Data Availability Statement

All datasets generated for this study are included in the article/Supplementary Material.

## Ethics Statement

The animal study was reviewed and approved by Inter American University Institutional Animal Care and Use Committee and University of Florida Institutional Animal Care and Use Committee (UF IACUC protocol #20180884). All samples were received and analyzed under Federal Fish and Wildlife Permit #MA067116-2.

## Author Contributions

JC: conceptualization, methodology, formal analysis, investigation, writing—original draft, visualization, and project administration. DC: conceptualization, methodology, investigation, and writing—review and editing. AM-G: resources and writing—review and editing. TA-Z: investigation. AR-G: resources and investigation. IL: supervision, writing—review and editing, and funding acquisition. All authors contributed to the article and approved the submitted version.

## Conflict of Interest

The authors declare that the research was conducted in the absence of any commercial or financial relationships that could be construed as a potential conflict of interest.
