# Supplementary material for: Manual Collection and Semen Characterization in a West Indian Manatee (Trichechus manatus)
Source: Front Vet Sci. 2020 Oct 22;7:569993. doi: 10.3389/fvets.2020.569993 (PMC7642902; doi:10.3389/fvets.2020.569993)
Supplement: Supplementary file 1 [file Table_1.docx]

**Supplementary Table 1.** Motility parameters for each individual ejaculate sample.

| Ejaculate | Total (%) | Progressive (%) | Non-Progressive (%) | Immotile (%) |
| --- | --- | --- | --- | --- |
| 1 | 88.3 | 79 | 9.3 | 11.7 |
| 2 | 98.4 | 95.2 | 3.1 | 1.6 |
| 3 | 100 | 99.5 | 0.5 | 0 |
| 4 | 99.1 | 89.8 | 9.3 | 0.9 |
| 5 | 97.9 | 93.7 | 4.2 | 2.1 |
| 6 | 82.7 | 71 | 11.6 | 17.4 |
| 7 | 30.5 | 8.4 | 22.1 | 69.5 |
| Mean ± *SD* | 85.3 ± 25 | 76.7 ± 31.7 | 8.6 ± 7.2 | 14.7 ± 25 |
