# Supplementary material for: Manual Collection and Semen Characterization in a West Indian Manatee (Trichechus manatus)
Source: Front Vet Sci. 2020 Oct 22;7:569993. doi: 10.3389/fvets.2020.569993 (PMC7642902; doi:10.3389/fvets.2020.569993)
Supplement: Supplementary file 2 [file Table_2.docx]

**Supplementary Table 2.** Kinematic parameters analyzed by the SCA^®^ system for progressively motile sperm for each individual ejaculate sample. Kinematic parameters for all ejaculate samples are further subdivided by progression velocity subgroups (rapid, medium, and slow). Velocity parameters include VCL, VSL, and VAP with larger numbers representing higher sperm velocities about the measured pathway trajectory. Velocity ratio pathway parameters include LIN, STR, and WOB. Larger values for LIN and STR represent larger trajectories and higher velocities. High values for WOB represent small trajectories. Flagellar beat parameters include ALH and BCF. Larger values for ALH represent larger distances between peaks and troughs along the sperm head pathway. Values for BCF correspond to the number of flagellar beats, which is assumed to occur each time the curvilinear path crosses the average path. Values are presented as mean ± *SD*.

| Ejaculate | Velocity | VCL (µm/s) | VSL (µm/s) | VAP (µm/s) | LIN (%) | STR (%) | WOB (%) | ALH (µm) | BCF (Hz) |
| --- | --- | --- | --- | --- | --- | --- | --- | --- | --- |
| 1 | Rapid | 120.8 ± 23.5 | 96.2 ± 20.2 | 100.8 ± 19.6 | 80.9 ± 15.2 | 95.3 ± 5.1 | 84.5 ± 13.4 | 2.9 ± 1.6 | 8.8 ± 3.8 |
|  | Medium | 69.7 ± 12 | 61 ± 13.2 | 63.5 ± 12.5 | 87.8 ± 12.6 | 95.7 ± 5.1 | 91.5 ± 10.7 | 1.3 ± 0.7 | 8.6 ± 4.3 |
|  | Slow | 36.2 ± 6.5 | 32.7 ± 8.5 | 33.6 ± 8.1 | 89.8 ± 15.5 | 96.9 ± 4.6 | 92.3 ± 13.6 | 0.6 ± 0.5 | 4.6 ± 4.5 |
| 2 | Rapid | 136.4 ± 29.3 | 117.4 ± 30.5 | 123.1 ± 29.5 | 86.3 ± 13.4 | 95.1 ± 6 | 90.5 ±11 | 2.1 ± 1.5 | 7.3 ± 3.7 |
|  | Medium | 70.6 ± 13.3 | 64.6 ± 14.7 | 65.9 ± 14.3 | 92.2 ± 14.6 | 97.9 ± 4.3 | 94 ± 13.2 | 0.8 ± 0.9 | 2.6 ± 3.6 |
|  | Slow | 37.3 ± 5.6 | 33.8 ± 8.2 | 34.7 ± 7.2 | 90.5 ± 16.3 | 96.7 ± 6.9 | 92.9 ± 12 | 0.4 ± 0.7 | 1.1 ± 2.9 |
| 3 | Rapid | 140.8 ± 28.1 | 120.4 ± 31.4 | 126.4 ± 30.4 | 85.5 ± 13.9 | 95 ± 6.4 | 89.7 ± 11.1 | 2.2 ± 1.3 | 7.5 ± 3.5 |
|  | Medium | 73.1 ± 12 | 68.2 ± 13.5 | 69.2 ± 12.9 | 93.6 ± 12.6 | 98.4 ± 4 | 94.9 ± 10.6 | 0.7 ± 0.8 | 3.3 ± 4 |
|  | Slow | 36.6 ± 4.6 | 33.8 ± 7.4 | 34.2 ± 7.1 | 92.5 ± 15.8 | 98.8 ± 2.6 | 93.4 ± 14.6 | 0.3 ± 0.5 | 1.6 ± 3.7 |
| 4 | Rapid | 117.3 ± 26.7 | 103 ± 26.3 | 108 ± 25.8 | 88 ± 11.5 | 95.2 ± 5.9 | 92.3 ± 8.7 | 1.2 ± 0.6 | 16.1 ± 6.1 |
|  | Medium | 74.3 ± 12 | 67.8 ± 12.8 | 70.2 ± 12.4 | 91.4 ± 9.9 | 96.5 ± 4.6 | 94.5 ± 7.5 | 0.7 ± 0.4 | 11.6 ± 8 |
|  | Slow | 30.6 ± 10 | 28 ± 10.5 | 29 ± 10 | 91.3 ± 15.2 | 96.1 ± 6.8 | 94.4 ± 12.3 | 0.2 ± 0.3 | 3.2 ± 5.5 |
| 5 | Rapid | 146.3 ± 34.8 | 136.3 ± 32.8 | 138.7 ± 32.5 | 93.6 ± 9.9 | 98.1 ± 3.2 | 95.2 ± 8.5 | 1.1 ± 0.7 | 17.6 ± 7.3 |
|  | Medium | 74.2 ± 10.1 | 68.6 ± 13.5 | 70.4 ± 11.6 | 92.3 ± 11.7 | 96.9 ± 5.7 | 94.9 ± 8.1 | 0.5 ± 0.5 | 7.3 ± 9 |
|  | Slow | 36.2 ± 5.5 | 32.3 ± 5.7 | 34 ± 5.4 | 89.7 ± 10.9 | 95 ± 4.2 | 94.3 ± 8.6 | 0.4 ± 0.4 | 7.1 ± 6.1 |
| 6 | Rapid | 116.2 ± 20.8 | 110.1 ± 21.9 | 112.5 ± 21.7 | 94.7 ± 7.8 | 97.8 ± 3.6 | 96.7 ± 6.2 | 0.9 ± 0.5 | 16.1 ± 5.9 |
|  | Medium | 70.7 ± 12.7 | 64.8 ± 14.3 | 66.8 ± 14 | 91.5 ± 10.2 | 96.9 ± 3.5 | 94.3 ± 8.7 | 0.8 ± 0.3 | 14.5 ± 5.9 |
|  | Slow | 34.8 ± 5.7 | 28.8 ± 7.2 | 30.8 ± 6.9 | 82.6 ± 15.9 | 92.9 ± 5.6 | 88.4 ± 14.3 | 0.6 ± 0.2 | 11.8 ± 6.7 |
| 7 | Rapid | 132.1 ± 24.3 | 129.8 ± 25 | 130.5 ± 24.8 | 98.1 ± 1.4 | 99.4 ± 0.3 | 98.7 ± 1.3 | 0.9 ± 0.2 | 18.2 ± 1.4 |
|  | Medium | 64.5 ± 13.6 | 60 ± 15.3 | 61.9 ± 14.1 | 92.3 ± 8.3 | 96.3 ± 5.4 | 95.7 ± 3.9 | 0.8 ± 0.2 | 17.8 ± 5.3 |
|  | Slow | 31.8 ± 5.3 | 24.8 ± 6.7 | 27.4 ± 6.5 | 77.7 ± 14.6 | 90 ± 5 | 85.8 ± 13.1 | 0.7 ± 0.1 | 14.2 ± 4.3 |
| Mean | Rapid | 132 ± 30 | 114.5 ± 30.7 | 119.8 ± 30 | 87 ± 13.1 | 95.3 ± 6 | 91 ± 10.5 | 1.8 ± 1.3 | 10.7 ± 6.4 |
|  | Medium | 72.4 ± 12.3 | 65.8 ± 13.6 | 67.9 ± 13.1 | 91.1 ± 11.6 | 96.8 ± 4.6 | 93.9 ± 9.5 | 0.8 ± 0.6 | 9.2 ± 7.4 |
|  | Slow | 33.3 ± 8 | 29.2 ± 9.1 | 30.6 ± 8.7 | 87.4 ± 16 | 94.9 ± 6.3 | 91.5 ± 13.4 | 0.4 ± 0.4 | 6.6 ± 7.1 |
|  | Rapid | 90.1 – 388.7 | 28 – 359.1 | 30.7 – 359.7 | 24.3 – 100 | 75 – 100 | 25.9 – 100 | 0 – 9.2 | 0 – 37 |
| Range | Medium | 45.1 – 90 | 19.9 – 89.9 | 20.3 – 89.9 | 26.2 – 100 | 75.2 – 100 | 26.7 – 100 | 0 – 3.8 | 0 – 31.8 |
|  | Slow | 11.9 – 44.9 | 4 – 44.3 | 4 – 44.3 | 27.3 – 100 | 75.3 – 100 | 30.2 – 100 | 0 – 2 | 0 – 26 |
