# Supplementary material for: Manual Collection and Semen Characterization in a West Indian Manatee (Trichechus manatus)
Source: Front Vet Sci. 2020 Oct 22;7:569993. doi: 10.3389/fvets.2020.569993 (PMC7642902; doi:10.3389/fvets.2020.569993)
Supplement: Supplementary file 3 [file Table_3.docx]

**Supplementary Table 3.** Percentage of normal and abnormal sperm morphologies of each individual ejaculate sample. Sperm morphology was visually assessed using an eosin-nigrosin stain.

|  | Morphological Parameters | | | | | | |
| --- | --- | --- | --- | --- | --- | --- | --- |
| Ejaculate | Normal (%) | Abnormal head (%) | Detached head (%) | Proximal droplet (%) | Distal droplet (%) | Abnormal midpiece (%) | Coiled/bent tail (%) |
| 1 | 21.5 | 0.5 | 32 | 0.5 | 1.5 | 15 | 30 |
| 2 | 39 | 1.5 | 15.5 | 0.5 | 3 | 14.5 | 28.5 |
| 3 | 52.5 | 2.5 | 24 | 0 | 1.5 | 7.5 | 15 |
| 4 | 49.5 | 1.5 | 13 | 2 | 0 | 11 | 24 |
| 5 | 57.5 | 2.5 | 7 | 0.5 | 2 | 15.5 | 18 |
| 6 | 55.5 | 3 | 11.5 | 2 | 1.5 | 12 | 18 |
| 7 | 54.5 | 2 | 9 | 2 | 0 | 8.5 | 7 |
| Mean ± *SD* | 47.1 ± 12.8 | 1.9 ± 0.8 | 16 ± 8.9 | 1.1 ± 0.9 | 1.4 ± 1.1 | 12 ± 3.2 | 20.1 ± 8.1 |
