# Supplementary material for: Manual Collection and Semen Characterization in a West Indian Manatee (Trichechus manatus)
Source: Front Vet Sci. 2020 Oct 22;7:569993. doi: 10.3389/fvets.2020.569993 (PMC7642902; doi:10.3389/fvets.2020.569993)
Supplement: Supplementary file 4 [file Table_4.DOCX]

**Supplementary Table 4.** Sperm characteristics for each individual ejaculate sample. Sperm characteristics included plasma membrane integrity, acrosome integrity, chromatin condensation, and chromatin maturation. Sperm characteristics were visually assessed by eosin-nigrosin, Coomassie blue, toluidine blue, and aniline blue-eosin stains, respectively.

| Ejaculate | Plasma membrane integrity (%) | Acrosome Integrity (%) | Chromatin Condensation (%) | Chromatin Maturation (%) |
| --- | --- | --- | --- | --- |
| 1 | 39.4 | 76 | 2 | 0 |
| 2 | 58.4 | 87 | 4 | 0 |
| 3 | 56.6 | 86 | 6 | 0 |
| 4 | 38.1 | 77 | 4.5 | 0 |
| 5 | 36.7 | 76 | 14 | 0.5 |
| 6 | 38.9 | 74 | 11.5 | 0 |
| 7 | 43.8 | 82.5 | 6 | 0 |
| Mean ± *SD* | 45.1 ± 10.3 | 79.8 ± 5.7 | 6.9 ± 4.3 | 0.1 ± 0.2 |
